# Integrating Phenotypic and Gene Expression Linkage Mapping to Dissect Rust Resistance in Chickling Pea

**DOI:** 10.3389/fpls.2022.837613

**Published:** 2022-04-07

**Authors:** Carmen Santos, Davide Coelho Martins, María José González-Bernal, Diego Rubiales, Maria Carlota Vaz Patto

**Affiliations:** ^1^Instituto de Tecnologia Química e Biológica António Xavier, Universidade Nova de Lisboa, Oeiras, Portugal; ^2^Institute for Sustainable Agriculture, Consejo Superior de Investigaciones Científicas, Córdoba, Spain

**Keywords:** quantitative trait loci-QTL, expression QTL-eQTL, *Lathyrus cicera*, *Uromyces pisi*, partial resistance, QTL hotspots

## Abstract

Rusts are among the most important foliar biotrophic fungal diseases in legumes. *Lathyrus cicera* crop can be severely damaged by *Uromyces pisi*, to which partial resistance has been identified. Nevertheless, the underlying genetic basis and molecular mechanisms of this resistance are poorly understood in *L. cicera*. To prioritise the causative variants controlling partial resistance to rust in *L. cicera*, a recombinant inbred line (RIL) population, segregating for response to this pathogen, was used to combine the detection of related phenotypic- and expression-quantitative trait loci (pQTLs and eQTLs, respectively). RILs’ *U. pisi* disease severity (DS) was recorded in three independent screenings at seedling (growth chamber) and in one season of exploratory screening at adult plant stage (semi-controlled field conditions). A continuous DS range was observed in both conditions and used for pQTL mapping. Different pQTLs were identified under the growth chamber and semi-controlled field conditions, indicating a distinct genetic basis depending on the plant developmental stage and/or the environment. Additionally, the expression of nine genes related to *U. pisi* resistance in *L. cicera* was quantified for each RIL individual and used for eQTL mapping. One *cis*-eQTL and one trans-eQTL were identified controlling the expression variation of one gene related to rust resistance – a member of glycosyl hydrolase family 17. Integrating phenotyping, gene expression and linkage mapping allowed prioritising four candidate genes relevant for disease-resistance precision breeding involved in adaptation to biotic stress, cellular, and organelle homeostasis, and proteins directly involved in plant defence.

## Introduction

*Lathyrus cicera* L. (chickling pea) is a dual-purpose cool season legume and a source of protein for animals (Hanbury et al., 2000) and humans (Peña-Chocarro and Peña, 1999) nutrition. This species-genus belongs to the tribe Fabeae (syn. Vicieae) along with *Vicia*, *Lens*, *Pisum*, and *Vavilovia* (reviewed in Smýkal et al., 2011). *Lathyrus sativus* (grass pea) is the most closely related relative to *L. cicera* and these two *Lathyrus* species have a close phylogenetic relationship with pea (*Pisum sativum*), so close that there are suggestions that the genus *Pisum* should be included in the genus *Lathyrus* (Schaefer et al., 2012).

*Lathyrus cicera* owns important agronomic traits as resistance to biotic and abiotic stresses (Vaz Patto and Rubiales, 2014; Hammer et al., 2019; Lambein et al., 2019). It is therefore an attractive choice for sustainable feed and food production, mainly in more marginal environments, and because of pathogen sharing, it could act as a promising alternative source of resistance to related species, such as grass pea and pea (Vaz Patto et al., 2006; Vaz Patto and Rubiales, 2014; Hammer et al., 2019).

Fungal diseases are major constraints for yield stability in legumes (Rubiales et al., 2015; Martins et al., 2020). Rusts are among the most important diseases recorded in grain and forage legumes (Sillero et al., 2006; Rubiales et al., 2011). Several rust species can infect legumes, most of them belonging to the *Uromyces* genus (Rubiales et al., 2011). A good example is the wide host range biotrophic *Uromyces pisi* that infects species of *Lathyrus*, *Pisum*, *Lens*, and *Vicia* genera (Farr, D.F., and Rossman, A.Y. Fungal Databases) (Barilli et al., 2012). Chemical control of rust is possible (Emeran et al., 2011), but the use of host plant resistance is the most economical and ecological desired means of control (Rubiales et al., 2011). Due to the reduced selective pressure imposed on the pathogen, the use of plant partial resistance is a potentially more durable approach than complete resistance (McDonald and Linde, 2002; Niks and Rubiales, 2002). Indeed, rusts are among the pathogens with the highest risk of breaking down the effectiveness of major resistance genes (R-genes) due to their effective air dispersal and the coexistence of sexual and asexual reproduction cycles (McDonald and Linde, 2002).

Partial resistance not associated with host cell necrosis (hypersensitive reaction) is common in major grain legumes against rusts (Sillero et al., 2006) and was already identified in *Lathyrus* spp. (Vaz Patto and Rubiales, 2009; Vaz Patto et al., 2009) or in *Pisum* spp. (Barilli et al., 2009a,b,c, 2012) against *U. pisi* and *U. vicia-fabae*. In the particular case of *Pisum fulvum*, a wild relative of pea, one to three quantitative trait loci (QTLs) controlling partial resistance to *U. pisi* have been identified (Barilli et al., 2010, 2018). In contrast, little is known about the genetic control of *L. cicera* resistance to *U. pisi*.

In an Iberian collection of *L. cicera* accessions, microscopic and macroscopic variable levels of resistance were identified against *U. pisi*. Resistant accessions partially restricted the formation of haustoria, resulting in a high percentage of early aborted fungal colonies, a decreased number of haustoria per colony, and a reduced intercellular growth of infection hyphae compared to susceptible accessions (Vaz Patto et al., 2009). A segregating recombinant inbred line (RIL) population was developed from the cross of the most contrasting *L. cicera* accessions of this Iberian collection. The RILs were later on used for the development and refinement of the first *L. cicera* linkage map with transcriptome based SNPs and e-SSR markers (retrieved from the RIL parental lines leaf RNAseq response to *U. pisi* infection) as well as genotype-by-sequencing based markers (Santos et al., 2018, 2020). The mentioned *L. cicera* RIL parental lines transcriptomic study also highlighted upregulated genes in response to rust infection involved in hormone metabolism, cell wall degradation, secondary metabolism, ROS production, signalling and regulation of transcription of defence (Santos et al., 2018). In spite of these recent efforts, the causative variants controlling partial resistance to *U. pisi* remain elusive, leading to a poor understanding of the partial genetic resistance and molecular mechanisms of *Lathyrus* spp. against rust disease.

Classical approaches to unveil quantitative resistance genetic basis in non-model species include QTL mapping in segregating populations and syntenic analyses using model/related species to search for orthologous genes within the QTL detected regions. Whereas much progress has been made in plant QTL mapping controlling phenotypic natural variation, this approach has been hampered by the complex interrelation of genetic variants and expression regulators (Hansen et al., 2008; Wallace et al., 2013; Albert and Kruglyak, 2015). In species with no sequenced or no fully assembled genome, as the *Lathyrus* spp. (Emmrich et al., 2020), alternative approaches may add value over classical QTL mapping. Genetical-genomics is considered a very powerful tool to improve our knowledge on the genetic architecture of complex traits, including disease response (Lima et al., 2018; Fauteux et al., 2019). In this approach, transcript expression levels are treated as quantitative phenotypes in a segregating population and the genomic variants that influence expression levels of each transcript are identified by conventional QTL analysis (Li and Burmeister, 2005). The found genomic regions controlling gene expression are referred to as expression-QTLs (eQTLs). Previous studies have reported that distant or trans-eQTLs may explain a higher proportion of expression variance than local (at the same locus as the structural gene) or *cis*-eQTLs (Liu et al., 2017; Carrasco-Valenzuela et al., 2019; Fauteux et al., 2019; Miculan et al., 2021). Hotspots of trans-eQTL may act as key regulators of phenotypes, whereas *cis*-eQTLs display local gene expression regulation, with co-regulated gene clusters (Wang et al., 2018; Miculan et al., 2021). Several studies have been using a hybrid approach (pQTL and eQTL analyses) to better understand the gene networks underlying traits of interest in plants (Lima et al., 2018; Carrasco-Valenzuela et al., 2019; Fauteux et al., 2019; Miculan et al., 2021).

The main aim of this work was to elucidate the genetic basis and putative molecular strategies of chickling pea partial resistance against rust disease. Using *L. cicera* RIL population phenotypic response to *U. pisi* infection (mainly at the seedling stage, under controlled growth chamber, and complemented by an exploratory assay at the adult plant stage, under semi-controlled field conditions), in the expression analysis of genes related to rust resistance and genomic data, we performed a combined pQTL/eQTL linkage mapping analysis. This will allow prioritising candidate genes for validation and future use in precision breeding, and advancing our understanding on the molecular mechanisms underlying partial resistance to *U. pisi* in *L. cicera*.

## Materials and Methods

### Plant Material

A segregating population of 103 F_6_ RILs, derived by single seed descendent from a cross between *L. cicera* genotypes BGE023542 and BGE008277, was repetitively phenotyped in response to rust (*U. pisi*) infection under controlled growth chamber conditions at the seedling stage and under semi-controlled field conditions at the adult plant stage.

The two parental genotypes showed contrasting phenotypes to *U. pisi* infection in an exploratory growth chamber condition (seedling stage) screening of Iberian germplasm (Vaz Patto et al., 2009). BGE023542 was partial resistant [Disease Severity (DS) = 36%, scored as the percentage of leaf area coverage by rust pustules; and Infection Type (IT) = 4 representing a compatible interaction with well-formed pustules with no associated chlorosis or necrosis (Stakman et al., 1662)], and BGE008277 susceptible (DS = 80%, IT = 4) (Vaz Patto et al., 2009).

### Chickling Pea Phenotypic Response Evaluation Against *Uromyces pisi*

Phenotypic response of *L. cicera* RIL individuals and parental lines to rust disease were studied at the seedling stage under a controlled growth chamber and the adult plant stage in one season exploratory experiment, under semi-controlled field conditions. *L. cicera* RIL individuals were inoculated with *U. pisi* monosporic isolate Up-CO-01, derived from a rust population earlier collected on pea fields at Córdoba and stored at Institute for Sustainable Agriculture-CSIC at –80°C. Prior to use for the screenings, rust spores were retrieved from the store and multiplied on the susceptible pea cv. “Messire.”

#### Adult Plant Stage Semi-Controlled Field Evaluations

The *L. cicera* RIL individuals and their parental genotypes were sown in Córdoba during the 2018/2019 growing season, under a tunnel covered with insect-proof net and drop irrigation. Five seeds per genotype were sown on 19 November 2018 in a one-row plot, with three plot repetitions (15 plants in total), using an alfa-lattice design. On the day of field inoculation, the conserved spores were heat-shocked at 40°C for 5 min and then diluted in a Tween-20 aqueous solution (0.03%, v:v), used as a wetting agent. Three-month-old seedlings were spray-inoculated at the sunset to benefit from darkness and higher relative humidity of the night promoting spore adhesion and germination. At plant maturity (6 months old and 3 months after inoculation), DS and IT were assessed. DS was visually estimated as the percentage of canopy covered by rust pustules. IT was assessed using the 0–4 scale of Stakman et al. (1962), where IT 0 = no symptoms, IT 1 = necrotic halo surrounding minute pustules barely sporulating; IT 2 = necrotic halo surrounding small pustules, IT 3 = chlorotic halo, and IT 4 = well-formed pustules with no associated chlorosis or necrosis.

#### Seedling Stage Growth Chamber Evaluations

The response of the *L. cicera* RIL population was also evaluated at the seedling stage under controlled growth chamber conditions, in three independent inoculation assays (assay repetitions). For each inoculation assay, five seedlings per RIL were grown in pots (one plant per pot), containing 250 cm^3^ of 1:1 sand-peat mixture, in a growth chamber at 20°C with a 12 h light/12 h dark photoperiod. Twenty-day-old seedlings were dust-inoculated with *U. pisi* spores diluted in pure talk (1:10) with the help of a small manual-dusting device. After inoculation, seedlings were incubated for 24 h at 20°C in complete darkness and 100% relative humidity and then transferred to a growth chamber at 20°C with a 12 h light/12 h dark photoperiod. The response to rust inoculation was assessed 11 days after inoculation by measuring DS and IT.

### Gene Expression Analysis

#### RNA Isolation, Quantification, and cDNA Synthesis

For gene expression quantification, leaves of the parental lines and each F_5_ RIL individual were inoculated with *U. pisi* Up-CO-01 under growth chamber conditions. One to three biological replicates of 15-days-old *L. cicera* seedlings were inoculated using the same procedure described previously. Inoculated leaves were collected at 37 h after inoculation (hai) to be consistent with the time-point used in the already mentioned *L. cicera* RIL parental lines rust response transcriptomic studies (Santos et al., 2018). This time-point corresponds to the infection stage between fungus growth prior to stoma penetration and the early stages of infection, till colony development and if applicable, the presence of host cell necrosis observed at a microscopic level (Vaz Patto and Rubiales, 2009). Collected leaves were immediately frozen in liquid nitrogen and stored at –80°C until RNA extraction. Total RNA was extracted from about 100 mg of inoculated leaves using the GeneJET Plant RNA Purification Mini Kit (Thermo Scientific, Vilnius, Lithuania), according to the manufacturer’s instructions. The extracted RNA was treated with Turbo DNase I kit (Ambion, Austin, TX, United States), according to the manufacturer’s instructions. RNA concentrations were measured by a Qubit 2.0 Fluorometer (Invitrogen, Life Technologies, Carlsbad, CA, United States) using a Qubit dsRNA BR Assay kit. The RNA purity was checked by measuring the ratios of absorbance at 260/280 nm and 230/280 nm using a NanoDrop device (Thermo Scientific, Passau, Germany).

The cDNA was synthesised from 1.5 μg of total RNA from each sample following the manufacturer’s instructions from the iScript cDNA Synthesis Kit (Biorad, Hercules, CA, United States).

#### Selection of Genes Related to Rust Resistance for Recombinant Inbred Line Expression Analysis

Differentially expressed genes (DEGs) related to rust resistance were selected from the *L. cicera* RIL parental lines RNAseq leaf-transcriptome in response to *U. pisi* infection previously obtained by Santos et al. (2018). The gene selection criteria were: (1) non-redundant DEGs with the log_2_ of the ratio between BGE023542 (partially resistant) and BGE008277 (susceptible) inoculated reads higher than 2 and lower than –2; (2) DEGs not directly involved in specific resistance mechanisms, such as oxidative, metabolic, and transporter activities; (3) DEGs involved in defence response and categorised in pathogen recognition, antifungal proteins, cell wall modification proteins or involved in the regulation of other defence related processes and (4) nucleotide sequences of DEGs suitable for primer design, as described in the next section “*Primer design*” and resulting in a single amplification (primer specificity).

Differentially expressed gene annotation was revised and updated from the previous study (Santos et al., 2018) by BLASTn search against the pea reference genome v1a^1^ (Kreplak et al., 2019) and against genomic sequences of other legume species deposited at NCBI databases. The molecular function and biological process from each DEG related to rust resistance were also investigated using InterPro (Hunter et al., 2009) and UniProt^2^ databases.

#### Primer Design

Primers were designed for the selected DEGs using as a template the gene sequence obtained by the JBrowse tool at https://urgi.versailles.inra.fr/Species/*Pisum*. The Primer3Plus tool^3^ (Boston, MA, United States) was used for primer design, with the default setting for Reverse Transcribed quantitative PCR (RT-qPCR) optimal conditions. Primer specificity was predicted using the Primer-BLAST NCBI tool (National Center for Biotechnology Information, United States), using the legume genomes deposited at NCBI. Specific primers were preferably designed in the 3′ intra-exonic regions and were synthesised by STABVida (Caparica, Portugal) (Supplementary Table 1).

#### Expression Analysis by Reverse Transcribed Quantitative PCR

The relative gene expression of selected DEGs was analysed by RT-qPCR on a Light Cycler^®^ 480 System, using the LightCycler^®^ 480 SYBR Green I Master protocol. PCR amplification efficiencies were tested for all primers for target and reference genes using cDNA two-fold dilution series. As reference genes, β-tubulin, photosystem I P700 apoprotein A2, γ-tubulin, chromodomain helicase DNA-binding protein, and histone H2A.2, previously described by Almeida et al. (2015) and Santos et al. (2018), were tested. Using the geNorm and NormFinder software packages from the GenEx v.5 software (MultiD, Goteborg, Sweden), two reference genes were selected for the gene relative expression analysis. Thermo cycling reactions were carried out following the described conditions: denaturation step at 90°C for 5 min; 45 cycles of amplification at 95°C for 10 s; 10 s at 60°C and 10 s at 72°C. For each reaction, a melting curve (dissociation stage) was performed to detect non-specific PCR products and/or contaminants. A non-template control (NTC), without cDNA, was also included for each primer mix to detect possible contaminations.

Relative expression levels (Fold change, FC) were calculated using the Pfaffl method (Pfaffl, 2001) compared with expression levels of the reference genes (β-tubulin and γ-tubulin) and using the susceptible parental line BGE008277 as a calibrator. Finally, FC data were transformed into a logarithmic scale (base 2) to meet the data normality assumptions for statistical analysis and graphical representation.

Since the number of biological replicates varied from 1 to 3, the absence of significant differences between biological replicates was confirmed by ANOVA using the Genstat software (Genstat^®^ for Windows 19th edition), considering the genotypes represented by three biological replicates. Therefore, the average of relative expression levels per RIL was used as a metric for eQTL detection.

### Phenotypic and Gene Expression Data Linkage Mapping Analysis

The descriptive statistical analyses of phenotypic DS data collected at the seedling stage under growth chamber and adult plant stage under semi-controlled field conditions, as well as of gene expression normalised data, were performed using the Genstat software (Genstat^®^ for Windows 19th edition). Graphical inspection of residuals was used to assess normality (Q-Q plot), homogeneity of variance (residuals versus fitted values), and to identify outliers. Observations exceeding 1.5 times the interquartile range were removed from the analysis. ANOVA was independently conducted for DS scored under growth chamber, semi-controlled field conditions, and gene expression normalised data, using the Genstat procedure. A *t*-test (*P* < 0.05) was used for means comparisons between relative expression of parental lines for each DEG under study. Broad-sense heritabilities, representing the percentage of the genetic variance in the total phenotypic variance, were calculated for phenotypic data using the VHERITABILITY Genstat procedure.

Phenotypic QTL (pQTL) and expression QTL (eQTL) linkage mapping analyses for rust resistance were performed using the MapQTL software version 5.0 (Van Ooijen, 2009). For pQTL analysis, the DS averages obtained across repetitions for each RIL under growth chamber or semi-controlled field conditions were used. The mean of the relative expression value of a gene in each RIL was treated as phenotypic data for the eQTL analysis. Interval mapping (Lander and Botstein, 1989) and multiple QTL mapping approaches (MQM) (Jansen and Stam, 1994) were applied. The significant LOD thresholds corresponding to a confidence level of *P* < 0.05 were estimated for each trait (DS scored under growth chamber and semi-controlled field conditions and gene expression data) using a permutation test with 1,000 permutations available in MapQTL software. Phenotypic QTLs and eQTLs were declared significant when LOD scores (MQM) exceeded the minimum significance LOD threshold. The coefficient of determination (*R*^2^) for the marker located at the pQTL/eQTL peak was used to estimate the percentage of the phenotypic/transcript abundance variance explained by the pQTL/eQTL. The 1-LOD support interval was also determined for each QTL LOD peak. The additive effect for each detected pQTL/eQTL was estimated using the MQM procedure.

Each significant pQTL/eQTL was characterised by the peak marker, the coefficient of determination (*R*^2^), LOD score, the QTL interval (including 1-LOD confidence support), and the additive effect. pQTL/eQTL representations were drawn using the MapChart 2.3 software (Voorrips, 2002). pQTL nomenclature was set as follows: *UpDSLG_chamber* and *UpDSLG_field*, where the “LG” was replaced by the number of the *L. cicera* map Linkage Group in which the pQTLs were detected for *U. pisi* under growth chamber and semi-controlled field conditions, respectively. eQTL nomenclature was set as the acronym of the gene followed by the LG, where the eQTLs for relative expression were detected. For pQTL/eQTLs mapped in the same LGs, those QTLs were distinguished with “a,” “b,” or “c” after the LG number, where “a” corresponds to the pQTL/eQTL with the highest LOD value and “c” to the pQTL/eQTL with the lowest LOD value. As an example, G*IucIVa* and *GlucIVb* referred to both eQTLs identified for relative expression of the *Gluc* gene detected on LGIV.

### Prediction of *Cis*- and *Trans*-Expression-Quantitative Trait Loci Through Syntenic Analysis With *Pisum sativum* Genome

The syntenic locations of *L. cicera* DEGs and eQTL intervals here studied were identified in the legume genomes publicly available to classify eQTLs into *cis* or *trans*. Using the DEGs and eQTL flanking markers’ nucleotide sequences, BLASTn tools were applied against *P. sativum* genome and the legume genomes deposited at NCBI (*e*-value < 1^–5^). The best BLAST hit obtained was used to define eQTLs as *cis* or *trans*-eQTLs. *Cis*-eQTLs were defined as being overlapping the transcribed region of the respective DEG (structural gene), while *trans*-eQTLs were defined as being located distant from the respective DEG (more than 1 Mbp) in the same chromosome or in a different chromosome. *L. cicera* DEGs and eQTL syntenic positions in the pea genome were represented and drawn using MapChart 2.3 software (Voorrips, 2002).

### Identification of Candidate Genes Underlying Phenotypic- and Expression-Quantitative Trait Loci

Candidate genes underlying pQTLs and eQTLs were predicted by the flanking markers’ sequence positional alignment to the legume sequence NCBI database and to the *P. sativum* reference genome v1a (Kreplak et al., 2019), using the BLASTn tool (*e*-value < 1^–5^). Flanking markers were defined as the closest markers to the boundaries of the QTL 1-LOD confidence interval (but still within the interval) with the possibility that only one marker (peak marker) was found within this interval. Additional candidate genes located within QTL regions were searched by comparative mapping of those pQTL/eQTL intervals in the pea genome, using the *P. sativum* Jbrowse platform^4^. The inferred position of the QTL intervals flanking markers in the pea genome was used to delimit the pea genome regions where to search for potential additional candidate genes.

## Results

### Rust Disease Severity Phenotypic Evaluation

**FIGURE 1:**
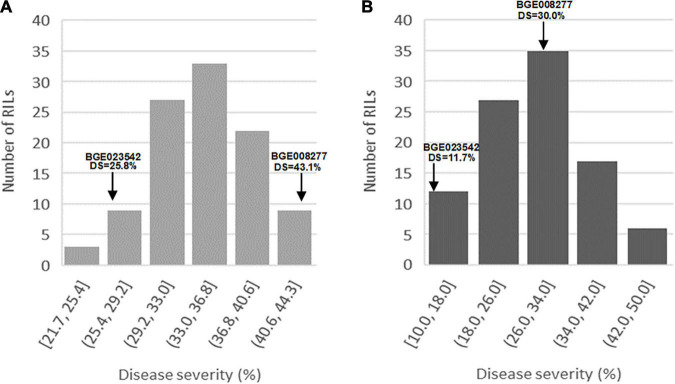
Frequency distributions of the *Lathyrus cicera* recombinant inbred line (RIL) population (BGE023542 × BGE008277) disease severity (DS) after inoculation with *Uromyces pisi*: **(A)** under growth chamber (controlled conditions) and **(B)** under semi-controlled field conditions. The average values of *U. pisi* DS of the two parental lines are indicated with arrows.

All *L. cicera* RIL individuals showed a compatible interaction (IT = 4) against *U. pisi* at the seedling stage under the growth chamber and at the adult plant stage under semi-controlled field conditions. This means that well-formed pustules with no associated macroscopically visible chlorosis or necrosis were observable on the leaf surface (Supplementary Figure 1). However, for both conditions, DS population frequency showed a continuous variation, ranging from 21.7 to 44.3% and from 10 to 50%, at the seedling stage under controlled and at adult plant stage under semi-controlled field conditions, respectively (Figure 1). The partially resistant parental accession BGE023542 showed a DS = 25.8 and 11.7% (IT = 4), at the seedling and adult plant stage, respectively (Figure 1). On the other hand, the susceptible parental accession BGE008277 showed a DS = 43.1% at the seedling and DS = 30% at the adult plant stage (Figure 1). Transgressive segregation was detected for DS against rust infection mainly under the adult plant stage, with a fraction of the individual RILs showing higher susceptibility than the susceptible parental genotype (BGE008277) (Figure 1). Little transgressive segregation was also observed at the seedling stage, with individual RILs more resistant than the partial resistant BGE023542 (Figure 1).

Since the residual’s variance followed a normal distribution for rust DS under growth chamber and semi-controlled field conditions, no data transformation was applied. Analysis of variance of rust DS revealed significant differences among the RIL individuals (*P* < 0.001) under both conditions (Supplementary Table 2). The calculated broad-sense heritability for rust DS across repetitions was similar at the seedling and adult plant stage, being 66.3 and 65.1%, respectively. Rust DS between seedling under growth chamber and adult plant stage under semi-controlled field conditions were positively, but weekly correlated (0.22, *P* < 0.05) (Table 1).

**TABLE 1:** Pearson’s correlation coefficients determined among rust disease severity (DS) and expression of genes related to rust resistance in the *Lathyrus cicera* RIL population (BGE023542 × BGE008277).

DS Growth Chamber	–									
**DS Field**	0.22*	–								
***Antifungal***	0.08	–0.15	–							
***CellSynt***	0.01	0.09	0.31**	–						
***Defence***	–0.08	–0.07	0.20	0.11	–					
***Extensin***	0.12	–0.05	0.48***	0.20	0.10	–				
***FKBP***	–0.04	0.12	0.31**	0.31**	0.04	0.35***	–			
***Gluc***	–0.06	–0.33**	0.30**	0.06	0.07	0.40***	0.09	–		
***LRR***	0.14	0.06	0.33**	0.35***	0.13	0.61***	0.39***	0.43***	–	
***MatE***	0.09	–0.04	–0.05	0.04	–0.12	0.07	–0.12	0.29**	0.24*	–
***Pi49***	0.18	–0.02	0.48***	0.30**	0.16	0.67***	0.29**	0.47***	0.69***	0.30**
	**DS Growth chamber**	**DS Field**	***Antifungal***	***CellSynt***	***Defence***	***Extensin***	***FKBP***	***Gluc***	***LRR***	***MatE***

P-values are indicated by asterisks as follows: *P < 0.05, **P < 0.01, and ***P < 0.001.

*Gene IDs: Antifungal, salt stress response/antifungal; CellSynt, cellulose synthase; Defence, defence response to bacterium + incompatible interaction; Extensin, proline rich extensin signature; FKBP, immunophilin precursor (FKBP15); Gluc, glycosyl hydrolases family 17; LRR, leucine rich repeat N-terminal domain; MatE, multidrug and toxic compound extrusion; Pi49, Pisum sativum disease resistance response protein.*

### Gene Expression Analyses

For the gene relative expression analysis, the β-tubulin and γ-tubulin were selected as reference genes, since both genes showed the most stable average expression among the reference genes tested.

**FIGURE 2:**
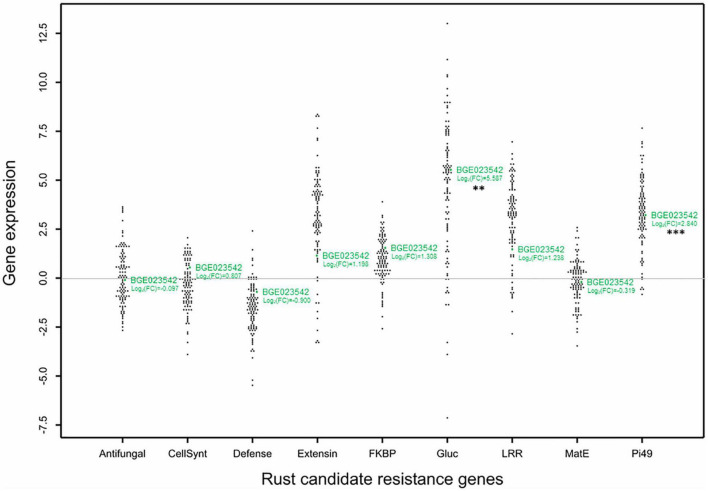
Dot plots histogram showing the distribution of relative expression (Log_2_ Fold change) of the selected genes related to rust partial resistance in the *Lathyrus cicera* recombinant inbred line (RIL) population (BGE023542 × BGE008277). The log_2_Fold Change values are represented in relation to the calibrator susceptible BGE008277 parental line, displayed by the horizontal line crossing *Y*-axis origin (log_2_ Fold Change = 0). Each black and green dot represents an RIL individual and the BGE023542 (partial resistant) parental line, respectively. *P*-values obtained from *t*-test calculated between relative expression of parental lines for each gene are indicated by asterisks as follows: ^**^P < 0.01 (*Gluc*) and ^***^P < 0.001 (*Pi49*).

Using the gene selection criteria previously defined, nine DEGs related to rust resistance were selected from the work of Santos et al. (2018; Table 2). For these genes, annotation was revised and updated from the original annotation (Santos et al., 2018; Table 2). The RIL individuals’ relative gene expression levels were measured for the nine selected DEGs and normalised to the mean of the susceptible parental line BGE008277. The relative gene expressions (evaluated as log_2_Fold Change) obtained among the *L. cicera* RIL population are depicted in comparative dot-histograms (Figure 2). Significant gene expression variation among the RIL individuals was observed for all genes under study (*P* < 0.001), being greater for the *Gluc* and *Extensin* genes (Figure 2). Nevertheless, when comparing the two RIL parental lines (partially resistant BGE023542 and the susceptible BGE008277), only two of the nine genes under study (*Pi49* and *Gluc*) showed significant differences (*P* < 0.05) (Figure 2). Moreover, significant correlations between gene expression and rust DS were only observed between DS under semi-controlled field conditions and *Gluc* relative expression (–0.33, *P* < 0.01) (Table 1).

**TABLE 2:** Genes related to rust resistance, selected from previously identified in a leaf-transcriptomic RNAseq study between BGE023542 (partially resistant) and BGE008277 (susceptible) *Lathyrus cicera* RIL parental lines in response to *Uromyces pisi* infection (Santos et al., 2018).

Reference assembly contig	BLAST best hit [Species/Gene or sequence ID/Chromosome position]	BLASTn *e*-value/% of similarity	Acronym	Differential expression (RT-qPCR)	Differential expression (RNA-seq from Santos et al., 2018)
					
a16587_204	Leucine rich repeat N-terminal domain [*Pisum sativum*, Psat7g094360 chr7LG7:155763094.155764871]	0.0/94%	*LRR*	1.24	4.03
a3776_385	*Pisum sativum* disease resistance response protein (PI49) [*Pisum sativum*, X13383.1, chr5LG3:279832614.279834259]	0.0/92%	*Pi49*	2.84	3.59
a103847_43	Cellulose synthase [*Pisum sativum*, Psat5g262200, chr5LG3:517072445.517075406]	0.0/96%	*CellSynt*	0.807	3.28
a9079_226	Glycosyl hydrolases family 17 [*Pisum sativum*, Psat7g17960, chr7LG7:337126816.337127902]	0.0/94%	*Gluc*	5.587	3.20
a8324_255	Salt stress response/antifungal [*Pisum sativum*, Psat0s66g0280, scaffold00066:107031.108584]	0.0/91%	*Antifungal*	–0.10	3.17
a1874_641	Proline rich extensin signature [*Pisum sativum*, Psat1g093960, chr1LG6:159014717.159016200]	1e-44/81%	*Extensin*	1.20	2.86
a4242_397	Immunophilin precursor (FKBP15) [*Vicia faba*, chr4 416511480.416514349]	0.0/93%	*FKBP*	1.31	2.85
a15929_195	Defence response to bacterium + incompatible interaction [*Pisum sativum*, Psat5g251880, chr5LG3:502621904.502623334]	0.0/89%	*Defence*	–0.90	2.42
a15672_145	Multidrug and toxic compound extrusion [*Pisum sativum*, Psat7g199240, chr7LG7:385262901.385269764]	0.0/95%	*MatE*	–0.32	3.20

*Differential expression (log_2_ Fold Change between parental lines) is shown using both the originally obtained RNA-seq data and using the present study RT-qPCR expression data. Reference assembly contigs are available in Santos et al. (2018).*

### Phenotypic Quantitative Trait Loci Mapping for Rust Disease Severity at Seedling and Adult Plant Stage

No significant differences were found between plot repetitions for DS under the semi-controlled field conditions (Supplementary Table 2). Although significant differences were found between inoculation assay repetitions for DS evaluated under growth chamber conditions (*P* < 0.001), the effect for genotype × inoculation assay interaction (*F* = 4.63) was much smaller than the genotype effect (*F* = 14.2). This supported the use of DS means across the three inoculation assays on the QTL analysis, increasing the power of QTL detection (Supplementary Table 2). Therefore, a univariate pQTL analysis was carried out using the means for DS obtained across repetitions under the semi-controlled field, as well as under the growth chamber conditions.

**FIGURE 3:**
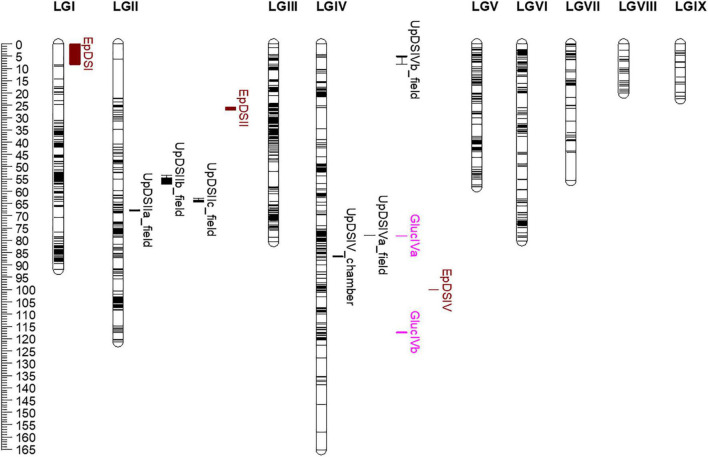
Phenotypic quantitative trait loci (pQTLs, in black) and expression QTLs (eQTLs, in pink) for rust (*Uromyces pisi*) inoculation response mapped on linkage groups (LG) of the high-density *Lathyrus cicera* genetic linkage map based on a recombinant inbred line population (BGE023542 × BGE008277) (Santos et al., 2020). Genetic distances given in centimorgans (Kosambi mapping function) are indicated by the ruler on the left. Horizontal black lines indicate marker positions along LGs. Boxes, extended by lines depicting the 1-LOD confidence interval, represent pQTL/eQTL intervals: in black pQTLs identified for *U. pisi* DS (disease severity [%]), under semi-controlled field conditions and growth chamber conditions; eQTLs are represented in pink (*GlucIVa* and *GlucIVb*). In brown are represented pQTL intervals for powdery mildew disease response (*Erysiphe pisi* and *E. trifolii*), previously identified in the same *L. cicera* RIL population (Santos et al., 2020).

Several and different pQTLs were identified for *U. pisi* response in the *L. cicera* RIL population at the seedling and adult plant stage. Five genomic regions associated with response to *U. pisi* DS at the adult plant stage under semi-controlled field conditions were mapped on LGII (*UpDSIIa_field*, *UpDSIIb_field*, and *UpDSIIc_field*) and on LGIV (*UpDSIVa_field* and *UpDSIVb_field*). On the other hand, only one pQTL was identified in response to *U. pisi* at the seedling stage under growth chamber conditions on LGIV–*UpDSIV_chamber* (Figure 3). pQTLs identified for *U. pisi* DS at the adult plant stage explained from 7.1 to 19% of the phenotypic variance observed (Table 3). The only detected *UpDSIV_chamber* QTL for *U. pisi* DS measured in seedlings under growth chamber conditions explained 10.7% of the phenotypic variance observed (Table 3). Resistant pQTL alleles (the ones contributing to a reduction in DS values) were derived from the partial resistant parental line (BGE023542) in all the pQTLs, except for the *UpDSIIa_field* QTL, where the resistant allele was derived from the most susceptible parental line BGE008277 (Table 3). This was also the strongest pQTL (based on LOD score, here 6.47), with the SSR LCI336 as peak marker (Table 3 and Figure 3). For each detected pQTL, besides the two flanking markers or peak marker, no other marker was found within the defined 1-LOD pQTL confidence intervals.

**TABLE 3:** Phenotypic quantitative trait loci (pQTLs) and expression QTLs (eQTLs) identified for response against *Uromyces pisi* in the *Lathyrus cicera* RIL population (BGE023542 × BGE008277).

Trait name^a^	QTL^b^	LG^c^	Peak QTL position (cM)	QTL interval (cM)^d^	LOD^e^	*R*^2^(%)^f^	Additive effect^g^
*U. pisi* DS (seedlings under growth chamber conditions)	*UpDSIV_chamber*	IV	86.885	86.372–86.885	2.52	10.7	–1.47
*U. pisi* DS (adult plants under semi-controlled field conditions)	*UpDSIIa_field*	II	68.043	67.567–68.043	6.47	19	6.60
	*UpDSIIb_field*	II	55.187	53.629–57.187	4.72	13.1	–3.2
	*UpDSIIc_field*	II	64.358	62.849–64.358	3.68	10.2	–4.88
	*UpDSIVa_field*	IV	77.983	77.865–77.983	5.86	16.9	–3.37
	*UpDSIVb_field*	IV	5.273	4.911–8.273	2.61	7.1	–2.14
*Gluc e*xpression (Log_2_FC)	*GlucIVa*	IV	78.252	78.19–78.252	9.29	32.2	1.94
	*GlucIVb*	IV	117.7	117.334–117.7	2.55	7.6	–0.95

*^a^U. pisi DS: disease severity (%) assessed after U. pisi inoculation in leaflets of L. cicera RIL under growth chamber and semi-controlled field conditions. Gluc gene expression: relative expression evaluated as Log_2_FC (Fold Change: Efficiency^∧–ΔΔCt^).*

*^b^Nomenclature assigned to QTL/eQTLs identified.*

*^c^LG, linkage group.*

*^d^pQTL/eQTL interval, including 1-LOD confidence support.*

*^e^LOD: the peak LOD score.*

*^f^R^2^: proportion of phenotypic/expression variance explained by the respective pQTL/eQTL (%).*

*^g^Additive effect = (mu_BGE023542 – mu_BGE008277)/2; mu_BGE023542: the estimated average of the distribution of the quantitative trait associated with the BGE023542 allele; mu_ BGE008277: idem for the BGE008277 allele. Negative or positive values indicate that favourable alleles came from BGE023542 or BGE008277, respectively.*

### Expression-Quantitative Trait Loci Mapping for the Expression of Genes Related to Rust Resistance After *Uromyces pisi* Infection

Glycosyl hydrolases family 17 (*Gluc*) was the only gene showing a significant (negative) correlation with DS and differential expression between the two RIL parental lines in the present study. Although *Pi49* expression also showed to be differential between parental lines, no significant correlation was observed with DS. Therefore, eQTL mapping was conducted only for the relative gene expression of *Gluc*, which led to the detection of two eQTLs, located on LGIV of the *L. cicera* linkage map (Figure 3 and Table 3). The eQTLs identified explained individually 7.6 (*GlucIVb*) and 32.2% (*GlucIVa*) of the gene expression variance observed (Table 3). The two eQTLs showed negative and positive additive effects (Table 3), indicating that alleles for increased gene expression came from both partial resistant and susceptible *L. cicera* RIL parental lines. The strongest eQTL (based on LOD score, here 9.29) was the *GlucIVa*, with the Silico DArT 100000355 as peak marker (Table 3). For each detected eQTL, besides the flanking markers or peak marker, no other marker was found within the defined 1-LOD eQTL confidence intervals.

By considering the predicted localisation of *L. cicera* eQTLs and differentially expressed gene sequences in the pea genome obtained by synteny analysis, we could define the detected eQTLs as one *cis*-eQTL and one trans-eQTLs. In particular, *GlucIVa* eQTL was located in the same locus of the *Gluc* structural gene (*cis*-eQTL) (Supplementary Figure 2). On the contrary, the *GlucIVb* eQTL was mapped in different chromosomes (considering the best BLAST hit against – against *M. truncatula* genome, Table 4) and distant to the structural gene (considering *P. sativum* genome) – *trans*-eQTL (Supplementary Figure 2).

**TABLE 4:** Phenotypic QTLs and expression QTLs’ flanking/peak markers and candidate genes identified for response to *Uromyces pisi* inoculation in the *Lathyrus cicera* RIL population (BGE023542 × BGE008277).

QTL	QTL flanking marker	Marker type	Blast hit [Species, gene ID, chromosome position]	Blast *e*-value/% similarity	Functions	References
*UpDSIV_ chamber*	c4_a65394	SNP	PPR repeat family [*Pisum sativum*, Psat7g133000, chr7LG7:223387290.223390628]	0.0/94%	Chloroplast-nucleus signalling pathway involved in in biotic and abiotic stresses	Lurin et al., 2004; Koussevitzky et al., 2007; Laluk et al., 2011; Xing et al., 2018

	100003641_52:C < T	SNP	Cyclin + N-terminal domain [*Pisum sativum*, Psat7g132800, chr7LG7:223222430.223229524]	1e-17/92%	Cell cycle and cell division	Wang et al., 2004

*UpDSIIa_field*	LCI336	SSR	Diacylglycerol kinase accessory domain [*Pisum sativum*, Psat4g140840, chr4LG4:277699073.277702768]	0.0/92%	Modulation of lipid signalling	Foka et al., 2020

	100003350	Silico Dart	No hits	–	–	–

*UpDSIIb_field*	39737826	Silico Dart	No hits	–	–	–

*UpDSIIc_field*	100000564	Silico Dart	Mitochondrial carrier protein signature [*Pisum sativum*, Psat1g049720, chr1LG6:77494954.77496860]	6E-21/98%	Metabolite transport across the mitochondrial inner membrane	Van Aken et al., 2009

	39732468a	Silico Dart	Utp21 specific WD40 associated putative domain [*Pisum sativum*, Psat1g051320 chr1LG6:80993359.81003124]	1e-16/91%	rRNA processing	Hunter et al., 2009

*UpDSIVa _field*	100000451	Silico Dart	No hits	–	–	–

	100002923_5:G > A	SNP	No hits	–	–	–

*UpDSIVb _field*	100000644	Silico Dart	No hits	–	–	–

	100000674	Silico Dart	No hits	–	–	–

*GlucIVa*	100000355	Silico Dart	No hits	–	–	–

	1000037810_21:A > G	SNP	Glycosyl hydrolases family 17 [*Pisum sativum*, Psat7g179640 chr7LG7:337132209.337133788	1e-10/90%	Hydrolyse 1,3-β-glucan polysaccharides plant and fungi cell wall matrix	Gaudioso-Pedraza and Benitez-Alfonso, 2014

*GlucIVb*	LCI220	SSR	Rust resistance kinase Lr10 [*Medicago truncatula*, LOC11443227], chr4:35653632.35671290]	0.0/91%	R-gene: coiled coil–nucleotide-binding site–leucine-rich repeat (CC–NBS–LRR)	Loutre et al., 2009

	100036350	Silico Dart	Protein SCO1 homologue 2 [*Medicago truncatula*, LOC25501925, chr8:42047078.42050300]	3e-07/85%	Participates in copper and redox homeostasis.	Attallah et al., 2011

*For each flanking/peak marker, the candidate gene ID and function, identified using BLASTn tools against the legume genomes publicly available, are shown.*

### Candidate Genes Underlying the Identified Phenotypic- and Expression-Quantitative Trait Loci

Potential candidate genes underlying the detected pQTLs and eQTLs associated with rust response in *L. cicera* RIL were inferred using BLASTn of nucleotide sequences from pQTL/eQTL intervals flanking or peak markers. Overall, identified candidate genes were predicted to be involved in cell cycle and division, adaptation to biotic stress, cellular and organelle homeostasis, mitochondrial redox, and proteins directly involved in plant defence (Table 4). In particular, when considering the *U. pisi* DS pQTLs, five candidate genes were identified: a pentatricopeptide repeat (PPR)-containing protein and a Cyclin + N-terminal domain at the seedling stage under growth chamber conditions; and a Diacylglycerol kinase, a Mitochondrial carrier, and a Utp21 specific WD40 associated putative domain, at the adult plant stage under semi-controlled field conditions (Table 4). The other three candidate genes were identified as underlying flanking markers of eQTLs for *Gluc* expression. Beyond the structural gene of *GlucIVa cis*-eQTL (Glycosyl hydrolases family 17), two more candidate genes were identified underlying the flanking markers of *GlucIVb trans*-eQTL: a rust resistance kinase Lr10 protein and a SCO1 homologue 2 (Table 4).

Additional candidate genes were searched within the pQTL/eQTL regions by comparative mapping of these intervals with the pea genome, using the syntenic flanking markers as delimitation of the genome windows where to search for potential candidate genes. Based on this approach, two syntenic regions were identified in pea for two of the detected pQTLs (*UpDSIV_chamber* and *UpDSIIc_field*). The candidate genes identified based on the comparative mapping are listed in Supplementary Table 3. Among all candidate genes found in homologous pea genome regions, some genes or gene families are known to be involved in host-pathogen interactions (Supplementary Table 3).

## Discussion

Most of the rust resistance reactions described so far in cool season legumes are incomplete and so potentially durable, but in most cases, the genetic basis of these resistances is still largely unknown, hampering their use in precision breeding (Rubiales et al., 2015). In this study, *L. cicera* RIL population segregating for rust resistance was used to elucidate the genetic basis and putative molecular strategies of chickling pea partial resistance against rust disease. By integrating the phenotypic response to *U. pisi* infection (at two different developmental plant stages and growing conditions), with the expression analysis of genes related to rust resistance and genomic data, we performed a combined pQTL/eQTL analysis. This allowed us to prioritise candidate genes that after validation, may be relevant for resistance precision breeding and advance our understanding of the molecular mechanisms underlying partial resistance to *U. pisi* in *L. cicera*.

All analysed *L. cicera* RIL individuals showed a compatible reaction with *U. pisi*, at the seedling stage, under a controlled growth chamber, as well as at the adult plant stage under semi-controlled field conditions in one-season exploratory analysis, characterised by well-formed pustules with no associated chlorosis or necrosis (IT = 4). As previously described in *L. cicera* (Vaz Patto et al., 2009), the most resistant genotypes presently identified, showed a low DS despite this compatible infection type, confirming their partial resistant nature (low DS, high IT) (McDonald and Linde, 2002; Niks and Rubiales, 2002). In the well-studied wheat-rust pathosystem, partial resistance commonly has a polygenic nature (controlled by adult plant resistance or APR genes), being expressed only in adult plants (except under very specific conditions) by a reduced and slow pathogen growth, without hypersensitive response. In contrast, pathogen race- or strain-specific major resistance genes (R-genes), generally conferring complete resistance, mostly function from seedling to adult growth stages (Dakouri et al., 2013; Ellis et al., 2014; Zegeye et al., 2014). In this study, DS evaluated in seedlings and adult plants were weakly correlated, indicating that also in *L. cicera* as in cereals, a different genetic basis, with multiple “minor effect” genes (explaining 7.1–19% observed variance) depending on the plant developmental stage may be involved in resistance. Indeed, it is widely acknowledged that partial resistance to rust is better identified in polycyclic infections, and even further, clearer on the adult plant stage than on seedlings (Sillero et al., 2006; Barilli et al., 2009a). This also seems to be the case in *L. cicera*. By considering even just one-season exploratory experiment at the adult plant stage, under semi-controlled field conditions, we allowed polycyclic infection vs. the monocyclic infection to occurr in the well-replicated growth chamber experiments at the seedling stage. Therefore, also in *L. cicera* there might be valuable small “adult plant” factors, not seen in the accurate monocyclic infections’ evaluations in seedlings. These findings are of utmost importance as currently, breeders put a higher emphasis on the discovery, characterisation, and complementary use of genes for partial, more durable resistance than on using only major effect R-genes, due to their potential lack of durability (Ellis et al., 2014).

From all the highlighted pQTL candidate genes, only the *PPR* (candidate for the *UpDSIV_chamber* pQTL), *DGK*, and *mitochondria carrier* genes *(UpDSIIa_field* and *UpDSIIc_field* pQTLs, respectively) were also identified differentially expressed between the RIL parental lines in the previous RNAseq *L. cicera-U. pisi* transcriptomic study (Fold Change of 0.94, 1.48, and 2.56, respectively) (Santos et al., 2018). For the remaining pQTL candidate genes, it was not possible to identify any DEG in the mentioned transcriptomic study, possibly due to the short nucleotide sequence available from DartSeq markers (65 bp), hampering a precise alignment between marker and transcriptomic sequences. Thus, the discussion of putative function in the variation of DS *L. cicera* response against *U. pisi* will focus on PPR, *DGK*, and *mitochondria carrier* genes, as the most promising candidate resistance genes considering the available information.

Pentatricopeptide proteins have been identified as playing important roles in organellar RNA metabolism, organ development, and in abiotic and biotic stresses (Lurin et al., 2004; Koussevitzky et al., 2007; Laluk et al., 2011; Xing et al., 2018). The function of PPR proteins has been reported in plant response to necrotrophic fungi and pathogenic bacteria (Laluk et al., 2011; Park et al., 2014). More recently, genes from the PPR gene family were identified underlying QTLs for partial resistance to the biotrophic *Erysiphe pisi* and *E. trifolii* powdery mildew pathogens in *L. cicera* (Santos et al., 2020) and in *L. sativus* (Martins et al., 2022^5^). All these studies reporting *PPR* as a gene involved in *Lathyrus* spp. response against biotrophic pathogens, instigate further analysis to the function of genes encoding for members of the *PPR* gene family on chickling pea response to rust fungi.

Concerning *DGK* genes, some studies have revealed their involvement in the modulation of plant growth and adaptation to both biotic and abiotic stresses. DGKs are the main moderators of lipid signalling in plants, and this enzymatic activity is increased upon pathogen infection or elicitor treatment in different species (Foka et al., 2020). In pea, for instance, the inhibition of DGK activity promoted an elicitor-mediated accumulation of the phytoalexin pisatin, inducing phenylalanine ammonia-lyase expression (Toyoda et al., 2000). In the present study, *UpDSIIa_field* pQTL (for which the *DGK* gene was proposed) showed a positive additive effect indicating that alleles for increased DS came from susceptible *L. cicera* parental line, and so, resistance may be caused by inhibition of DGK activity, as suggested for pea. However, in the previous *L. cicera* transcriptomic study (Santos et al., 2018), the partial resistant parental line showed higher expression of this *DGK* gene than the susceptible line (Fold Change of 1.48). Therefore, the involvement of *DGK* genes in the *L. cicera* response against *U. pisi* remains unclear.

The mitochondrial carrier protein family is over-represented among the stress-responsive genes, suggesting that stress induces altered needs for metabolite transport across the mitochondrial inner membrane (Van Aken et al., 2009). Mitochondrial carriers are highly expressed in stress conditions, such as application of cadmium or auxin, exposure to cold, and induction of cell death. Abscisic acid application, on the other hand, decreased the expression of some mitochondrial carriers (Millar and Heazlewood, 2003). The potential role of *L. cicera* mitochondrial carriers against *U. pisi* infection here indicated is in accordance with the previous transcriptomic study (Santos et al., 2018).

To better understand the complex interrelation of genetic variants and expression regulators, the identification of eQTLs for some genes related to *L. cicera* resistance against *U. pisi* was integrated in this study. From the initial nine genes selected from the transcriptomic data obtained for *L. cicera* RIL parental lines (BGE023542 and BGE008277) inoculated with *U. pisi* (Santos et al., 2018), *Gluc* (Glycosyl hydrolase family 17) was the only analysed gene which RILs’ expression levels correlated with RILs’ DS scorings in the present study. Glycosyl hydrolases family 17 serves diverse roles in plant defence and development, since it comprises degrading enzymes of 1,3-β-glucan polysaccharides found in the cell wall matrix of plants and fungi (Thomas et al., 2000; Gaudioso-Pedraza and Benitez-Alfonso, 2014). The significant negative correlation (*P* < 0.01) observed between DS at adult plant stage under semi-controlled field conditions and *Gluc* relative expression support that this gene expression may increase *L. cicera* resistance against *U. pisi*. Indeed, the partial resistant BGE023542 parental line showed higher transcript abundance than BGE008277 for this gene, suggesting that its expression may increase resistance to the pathogen. Positive and negative additive effects were found in the eQTLs for *Gluc*, indicating that both parental lines may harbour alleles for *L. cicera* resistance to rust.

Two eQTLs were detected for *Gluc* RIL expression variation. Strong eQTLs are typically *cis*-regulated (Võsa et al., 2021), and this was also observed in the present study as *GlucIVa* eQTL (the only detected cis-eQTL) showed the highest LOD score (9.29) and the highest percentage of explained expression variation (32.2%). Nevertheless, the influence of distal regulations is not trivial and generally numerous plant genes are controlled by distant-factors (Swanson-Wagner et al., 2009; Wang et al., 2010; Hammond et al., 2011; Cubillos et al., 2012). Indeed, a distant-eQTL (*trans*-eQTL) was detected in the present study associated with the expression variation of *Gluc* but explaining a smaller percentage of variation.

The candidate genes for the *GlucIVb* trans-eQTL were proposed to be a SCO1 protein and a rust resistance kinase Lr10. SCO1 protein has been described as playing a role in cellular copper homeostasis and mitochondrial redox signalling (Attallah et al., 2011). Regarding the Lr10 leaf rust resistance gene, in wheat encodes a coiled coil–nucleotide-binding site–leucine-rich repeat (CC–NBS–LRR) (Loutre et al., 2009). The majority of disease resistance genes (R-genes) isolated from plants, conferring resistance to pathogens, encode proteins containing an NBS–LRR domain, used for pathogen perception triggering host response (Jones and Dangl, 2006). To the best of our knowledge, no direct relation between glycosyl hydrolases and SCO1 and Lr10 genes were described so far. Therefore, further studies are crucial to unveil if and how the rust resistance kinase Lr10 and SCO1 may regulate Glycosyl hydrolases family 17 members in response to rust infection in *L. cicera*.

Noteworthy, the function and position of genes underlying the identified pQTLs and eQTLs should be confirmed when the scaffolding of the assembly of the *L. sativus* genome (Emmrich et al., 2020) to the pseudochromosome level and gene annotation becomes completed.

Disease resistance genes are commonly clustered in genetic regions, conferring resistance to different pathogens and/or to different races of the same pathogen (Michelmore and Meyers, 1998; Loridon et al., 2005). This has been previously reported in *Pisum* spp. for fungal and oomycete pathogens (Barilli et al., 2018; Jha et al., 2021; Wu et al., 2021). In *L. cicera*, a similar situation seems to occur to a certain extent. One of the recently identified QTLs for partial resistance to *E. pisi* (*EpDSIV* in Santos et al., 2020) locates within a close distance to pQTLs and eQTLs here identified for partial resistance to *U. pisi*: about 13.8 cM to the *UpDSIV_chamber*, 22 cM to the *UpDSIVa_field* and *GlucIVa*, and 17.5 cM to the *GlucIVb* eQTL. Therefore, a QTL hotspot for partial disease resistance to different biotrophic fungi (*U. pisi* and *E. pisi*) may be suggested in *L. cicera* LGIV (considering *UpDSIV_chamber, UpDSIVa_field*, *GlucIVa*, and *GlucIVb* and *EpDSIV;* Santos et al., 2020).

In the present study, only the *Gluc* and *Pi49* genes showed significant differences in gene expression by RT-qPCR between the two *L. cicera* parental lines. A motive that might be influencing our results is that different biological material was used on the RNAseq (a pool of equally mixed RNA individually extracted from 24 biological replicates per genotype) vs. the RT-qPCR (3 biological replicates individual RNA samples per genotype). Some residual heterozygosity potentially present in the RIL parental lines might have been exposed during different processing and contribute to the different fold changes (and consequently significant differences) obtained between gene expression of the RIL parental lines using RNA-seq and RT-qPCR techniques. The limited number of DEG from the RIL parental lines RNAseq data screened in the RIL individuals hampered the scope of the integration approach for prioritising candidate genes for chickling pea rust resistance. Nevertheless, the information obtained from the eQTL linkage mapping allowed a better understanding of the complex interrelation of genetic variants and expression regulators of the DEG *Gluc*.

## Conclusion

Different pQTLs for *U. pisi* partial resistance were identified in *L. cicera* RIL, suggesting that a different genetic control may be involved in different stages of plant development. The integration of these linkage mapping results and the previously obtained RIL parental lines transcriptomic data (Santos et al., 2018), helped to prioritise QTLs candidate genes (if DEG between the parental lines) as a cross-validation approach. This integration has materialised also by the selection of à *priori* genes related to resistance to rust from the RIL parental lines transcriptomic study (Santos et al., 2018) to “validate” at the RIL level by RT-qPCR. Taking all the above into consideration, the *L. cicera* response to *U. pisi* candidate gene prioritised list for future validation and use in precision breeding is constituted by *PPR*, *DGK*, *Mitochondrial carrier*, and *Gluc* genes. Furthermore, the presence of a putative hotspot of resistance-related genes in the *L. cicera* LGIV is suggested. Candidate genes underlying the identified pQTLs/eQTLs hotspot will be useful for a better understanding of the complex interrelation of genetic variants and regulators of expression related to the partial resistance of *L. cicera* against rust.

## Data Availability Statement

The original contributions presented in the study are included in the article/Supplementary Material, further inquiries can be directed to the corresponding author/s.

## Author Contributions

CS, DCM, and MJG-B contributed to phenotyping and methodology. CS contributed to gene expression analysis, QTL and eQTL analysis, software, and data curation. DR contributed to RIL population development. MCVP and CS contributed to conceptualisation, formal analysis, and writing—original draft preparation. CS, DCM, DR, and MCVP contributed to writing, reviewing, and editing. MCVP and DR contributed to funding acquisition, supervision, and resources. MCVP contributed to project administration. All authors have read and agreed to the published version of the manuscript.

## Conflict of Interest

The authors declare that the research was conducted in the absence of any commercial or financial relationships that could be construed as a potential conflict of interest.

## Publisher’s Note

All claims expressed in this article are solely those of the authors and do not necessarily represent those of their affiliated organizations, or those of the publisher, the editors and the reviewers. Any product that may be evaluated in this article, or claim that may be made by its manufacturer, is not guaranteed or endorsed by the publisher.

## Footnotes

1
https://urgi.versailles.inra.fr/blast/

2
www.uniprot.org

3
https://primer3plus.com

4
https://www.pulsedb.org/jbrowse/

5 Martins, D., Santos, C., Crespo, P., Sampaio, A. M., Rubiales, D., and Vaz Patto, M. C. (2022). Common and Unique Genetic Factors of Grass Pea (*Lathyrus sativus*) Oligogenic Partial Resistance Against two Powdery Mildew Pathogens (*Erysiphe pisi* and *Erysiphe trifolii)*.

## Supplementary Material

Supplementary Figure 1 Leaves of *Lathyrus cicera* inoculated with *Uromyces pisi*, showing a compatible reaction between plant and pathogen (IT = 4), **(A)** under controlled conditions (growth chamber) 11 days after inoculation with *U. pisi* and **(B)** under semi-controlled field conditions 3 months after inoculation with *U. pisi*.

Supplementary Figure 2 Expression QTLs syntenic regions of *Lathyrus cicera* recombinant inbred lines population (BGE023542 × BGE008277) for rust (*Uromyces pisi*) response mapped on the physical map of *Pisum sativum*. Physical distances given in Mbp are indicated for each *P. sativum* chromosome. Horizontal pink line indicates the syntenic position of *L. cicera* differentially expressed gene in *P. sativum* chromosomes. Black horizontal lines represent the syntenic regions of *L. cicera* eQTL intervals mapped on *P. sativum* genome.

## References

[B1] Albert F. W. Kruglyak L. (2015). The role of regulatory variation in complex traits and disease. *Nat. Rev. Genet.* 16 197–212. 10.1038/NRG3891 25707927

[B2] Almeida N. F. Krezdorn N. Rotter B. Winter P. Rubiales D. Vaz Patto M. C. (2015). *Lathyrus sativus* transcriptome resistance response to *Ascochyta lathyri* investigated by deepSuperSAGE analysis. *Front. Plant Sci.* 6:178. 10.3389/fpls.2015.00178 25852725 PMC4367168

[B3] Attallah C. V. Welchen E. Martin A. P. Spinelli S. V. Bonnard G. Palatnik J. F. (2011). Plants contain two SCO proteins that are differentially involved in cytochrome c oxidase function and copper and redox homeostasis. *J. Exp. Bot.* 62 4281–4294. 10.1093/JXB/ERR138 21543521

[B4] Barilli E. Cobos M. J. Carrillo E. Kilian A. Carling J. Rubiales D. (2018). A high-density integrated DArTseq SNP-based genetic map of *Pisum fulvum* and identification of QTLs controlling rust resistance. *Front. Plant Sci.* 9:167. 10.3389/fpls.2018.00167 29497430 PMC5818415

[B5] Barilli E. Moral A. Sillero J. C. Rubiales D. (2012). Clarification on rust species potentially infecting pea (*Pisum sativum* L.) crop and host range of *Uromyces pisi* (Pers.) Wint. *Crop Prot.* 37 65–70. 10.1016/j.cropro.2012.01.019

[B6] Barilli E. Satovic Z. Rubiales D. Torres A. M. (2010). Mapping of quantitative trait loci controlling partial resistance against rust incited by *Uromyces pisi* (Pers.) Wint. in a *Pisum fulvum* L. intraspecific cross. *Euphytica* 175 151–159. 10.1007/s10681-010-0141-z

[B7] Barilli E. Sillero J. C. Fernández-Aparicio M. Rubiales D. (2009a). Identification of resistance to *Uromyces pisi* (Pers.) Wint. in *Pisum* spp. germplasm. *Field Crops Res.* 114 198–203. 10.1016/j.fcr.2009.07.017

[B8] Barilli E. Sillero J. C. Moral A. Rubiales D. (2009b). Characterization of resistance response of pea (*Pisum* spp.) against rust (*Uromyces pisi*). *Plant Breed.* 128 665–670. 10.1111/j.1439-0523.2008.01622.x

[B9] Barilli E. Sillero J. C. Serrano A. Rubiales D. (2009c). Differential response of pea (*Pisum sativum*) to rusts incited by *Uromyces viciae-fabae* and *U. pisi*. *Crop Prot.* 28 980–986. 10.1016/j.cropro.2009.06.010

[B10] Carrasco-Valenzuela T. Muñoz-Espinoza C. Riveros A. Pedreschi R. Arús P. Campos-Vargas R. (2019). Expression QTL (eQTLs) analyses reveal candidate genes associated with fruit flesh softening rate in peach [*Prunus persica* (L.) Batsch]. *Front. Plant Sci.* 10:1581. 10.3389/fpls.2019.01581 31850046 PMC6901599

[B11] Cubillos F. A. Coustham V. Loudet O. (2012). Lessons from eQTL mapping studies: Non-coding regions and their role behind natural phenotypic variation in plants. *Curr. Opin. Plant Biol.* 15 192–198. 10.1016/j.pbi.2012.01.005 22265229

[B12] Dakouri A. McCallum B. D. Radovanovic N. Cloutier S. (2013). Molecular and phenotypic characterization of seedling and adult plant leaf rust resistance in a world wheat collection. *Mol. Breed.* 32 663–677. 10.1007/S11032-013-9899-8 24078786 PMC3782647

[B13] Ellis J. G. Lagudah E. S. Spielmeyer W. Dodds P. N. (2014). The past, present and future of breeding rust resistant wheat. *Front. Plant Sci.* 5:641. 10.3389/FPLS.2014.00641 25505474 PMC4241819

[B14] Emeran A. A. Sillero J. C. Fernández-Aparicio M. Rubiales D. (2011). Chemical control of faba bean rust *(Uromyces viciae-fabae)*. *Crop Prot.* 30 907–912. 10.1016/j.cropro.2011.02.004

[B15] Emmrich P. M. F. Sarkar A. Njaci I. Kaithakottil G. G. Ellis N. Moore C. (2020). A draft genome of grass pea *(Lathyrus sativus)*, a resilient diploid legume. *Biorxiv* 10.1101/2020.04.24.058164

[B16] Fauteux F. Wang Y. Rocheleau H. Liu Z. Pan Y. Fedak G. (2019). Characterization of QTL and eQTL controlling early *Fusarium graminearum* infection and deoxynivalenol levels in a Wuhan 1 x Nyubai doubled haploid wheat population. *BMC Plant Biol.* 19:536. 10.1186/s12870-019-2149-4 31795937 PMC6892237

[B17] Foka I. C. K. Ketehouli T. Zhou Y. Li X.-W. Wang F.-W. Li H. (2020). The emerging roles of Diacylglycerol Kinase (DGK) in plant stress tolerance, growth, and development. *Agronomy* 10:1375. 10.3390/AGRONOMY10091375

[B18] Gaudioso-Pedraza R. Benitez-Alfonso Y. (2014). A phylogenetic approach to study the origin and evolution of plasmodesmata-localized glycosyl hydrolases family 17. *Front. Plant Sci.* 5:212. 10.3389/FPLS.2014.00212 24904609 PMC4033164

[B19] Hammer K. Laghetti G. Direnzo P. Castelli A. Mikić A. (2019). Resources and opportunities for re-establishing *Lathyrus cicera* L. as a multipurpose cultivated plant. *Genet. Resour. Crop Evol.* 66 523–544. 10.1007/s10722-018-0717-3

[B20] Hammond J. P. Mayes S. Bowen H. C. Graham N. S. Hayden R. M. Love C. G. (2011). Regulatory hotspots are associated with plant gene expression under varying soil phosphorus supply in *Brassica rapa*. *Plant Physiol.* 156 1230–1241. 10.1104/pp.111.175612 21527424 PMC3135916

[B21] Hanbury C. White C. Mullan B. Siddique K. H. (2000). A review of the potential of *Lathyrus sativus* L. and *L. cicera* L. grain for use as animal feed. *Anim. Feed Sci. Technol.* 87 1–27. 10.1016/S0377-8401(00)00186-3

[B22] Hansen B. G. Halkier B. A. Kliebenstein D. J. (2008). Identifying the molecular basis of QTLs: eQTLs add a new dimension. *Trends Plant Sci.* 13 72–77. 10.1016/j.tplants.2007.11.008 18262820

[B23] Hunter S. Apweiler R. Attwood T. K. Bairoch A. Bateman A. Binns D. (2009). InterPro: the integrative protein signature database. *Nucleic Acids Res.* 37 D211–D215. 10.1093/nar/gkn785 18940856 PMC2686546

[B24] Jansen R. C. Stam P. (1994). High resolution of quantitative traits into multiple loci via interval mapping. *Genetics* 136 1447–1455. 10.1093/genetics/136.4.1447 8013917 PMC1205923

[B25] Jha A. B. Gali K. K. Alam Z. Lachagari V. B. R. Warkentin T. D. (2021). Potential application of genomic technologies in breeding for fungal and oomycete disease resistance in pea. *Agronomy* 11:1260. 10.3390/AGRONOMY11061260

[B26] Jones J. Dangl J. (2006). The plant immune system. *Nature* 444 323–329. 10.1038/nature05286 17108957

[B27] Koussevitzky S. Nott A. Mockler T. C. Hong F. Sachetto-Martins G. Surpin M. (2007). Signals from chloroplasts converge to regulate nuclear gene expression. *Science* 316 715–719. 10.1126/science. 17395793

[B28] Kreplak J. Madoui M.-A. Cápal P. Novák P. Labadie K. Aubert G. (2019). A reference genome for pea provides insight into legume genome evolution. *Nat. Genet.* 51 1411–1422. 10.1038/s41588-019-0480-1 31477930

[B29] Laluk K. AbuQamar S. Mengiste T. (2011). The *Arabidopsis* mitochondria-localized pentatricopeptide repeat rotein PGN unctions in defense against necrotrophic fungi and abiotic stress tolerance. *Plant Physiol.* 156 2053–2068. 10.1104/pp.111.177501 21653783 PMC3149943

[B30] Lambein F. Travella S. Kuo Y.-H. Van Montagu M. Heijde M. (2019). Grass pea (*Lathyrus sativus* L.): orphan crop, nutraceutical or just plain food? *Planta* 250 821–838. 10.1007/s00425-018-03084-0 30719530

[B31] Lander E. S. Botstein D. (1989). Mapping mendelian factors underlying quantitative traits using RFLP linkage maps. *Genetics* 121 185–199. 10.1093/genetics/121.1.185 2563713 PMC1203601

[B32] Li J. Burmeister M. (2005). Genetical genomics: combining genetics with gene expression analysis. *Hum. Mol. Genet.* 14 R163–R169. 10.1093/hmg/ddi267 16244315

[B33] Lima R. P. M. Curtolo M. Merfa M. V. Cristofani-Yaly M. Machado M. A. (2018). QTLs and eQTLs mapping related to citrandarins’ resistance to *Citrus gummosis* disease. *BMC Genomics* 19:516. 10.1186/s12864-018-4888-2 29969985 PMC6031180

[B34] Liu H. Luo X. Niu L. Xiao Y. Chen L. Liu J. (2017). Distant eQTLs and non-coding sequences play critical roles in regulating gene expression and quantitative trait variation in maize. *Mol. Plant* 10 414–426. 10.1016/J.MOLP.2016.06.016 27381443

[B35] Loridon K. McPhee K. Morin J. Dubreuil P. Pilet-Nayel M. L. Aubert G. (2005). Microsatellite marker polymorphism and mapping in pea (*Pisum sativum* L.). *Theor. Appl. Genet.* 111 1022–1031. 10.1007/s00122-005-0014-3 16133320

[B36] Loutre C. Wicker T. Travella S. Galli P. Scofield S. Fahima T. (2009). Two different CC-NBS-LRR genes are required for Lr10-mediated leaf rust resistance in tetraploid and hexaploid wheat. *Plant J.* 60 1043–1054. 10.1111/J.1365-313X.2009.04024.X 19769576

[B37] Lurin C. Andrés C. Aubourg S. Bellaoui M. Bitton F. Bruyère C. (2004). Genome-wide analysis of *Arabidopsis* pentatricopeptide repeat proteins reveals their essential role in organelle biogenesis. *Plant Cell* 16 2089–2103. 10.1105/tpc.104.022236 15269332 PMC519200

[B38] Martins D. De Sousa Araújo S. Rubiales D. Vaz Patto M. C. (2020). Legume crops and biotrophic pathogen interactions: a continuous cross-talk of a multilayered array of defense mechanisms. *Plants* 9:1460. 10.3390/plants9111460 33137969 PMC7692723

[B39] McDonald B. A. Linde C. (2002). Pathogen population genetics, evolutionary potential, and durable resistance. *Annu. Rev. Phytopathol.* 40 349–379. 10.1146/annurev.phyto.40.120501.101443 12147764

[B40] Michelmore R. W. Meyers B. C. (1998). Clusters of resistance genes in plants evolve by divergent selection and a birth-and-death process. *Genome Res.* 8 1113–1130. 10.1101/gr.8.11.1113 9847076

[B41] Miculan M. Nelissen H. Hassen M. Ben Marroni F. Inzé D. Pè M. E. (2021). A forward genetics approach integrating genome-wide association study and expression quantitative trait locus mapping to dissect leaf development in maize (*Zea mays*). *Plant J.* 107 1056–1071. 10.1111/TPJ.15364 34087008 PMC8519057

[B42] Millar A. H. Heazlewood J. L. (2003). Genomic and proteomic analysis of mitochondrial carrier proteins in *Arabidopsis*. *Plant Physiol.* 131 443–453. 10.1104/PP.009985 12586869 PMC166821

[B43] Niks R. E. Rubiales D. (2002). Potentially durable resistance mechanisms in plants to specialised fungal pathogens. *Euphytica* 124 201–216. 10.1023/A:1015634617334

[B44] Park Y. J. Lee H. J. Kwak K. J. Lee K. Hong S. W. Kang H. (2014). MicroRNA400-guided cleavage of pentatricopeptide repeat protein mrnas renders *Arabidopsis thaliana* more susceptible to pathogenic bacteria and fungi. *Plant Cell Physiol.* 55 1660–1668. 10.1093/pcp/pcu096 25008976

[B45] Peña-Chocarro L. Peña L. Z. (1999). History and traditional cultivation of *Lathyrus sativus* L. and *Lathyrus cicera* L. in the Iberian peninsula. *Veg. Hist. Archaeobot.* 8 49–52. 10.2307/23417642

[B46] Pfaffl M. W. (2001). A new mathematical model for relative quantification in real-time RT-PCR. *Nucleic Acids Res.* 29:e45. 10.1093/nar/29.9.e45 11328886 PMC55695

[B47] Rubiales D. Castillejo M. A. Madrid E. Barilli E. Rispail N. (2011). Legume breeding for rust resistance: lessons to learn from the model *Medicago truncatula*. *Euphytica* 180 89–98. 10.1007/s10681-011-0367-4

[B48] Rubiales D. Fondevilla S. Chen W. Gentzbittel L. Higgins T. J. V. Castillejo M. A. (2015). Achievements and challenges in legume breeding for pest and disease resistance. *Crit. Rev. Plant Sci.* 34 195–236. 10.1080/07352689.2014.898445

[B49] Santos C. Almeida N. F. Alves M. L. Horres R. Krezdorn N. Leitão S. T. (2018). First genetic linkage map of *Lathyrus cicera* based on RNA sequencing-derived markers: Key tool for genetic mapping of disease resistance. *Hortic. Res.* 5:45. 10.1038/s41438-018-0047-9 30181885 PMC6119197

[B50] Santos C. Martins D. Rubiales D. Vaz Patto M. C. (2020). Partial resistance against *Erysiphe pisi* and *E. trifolii* under different genetic control in *Lathyrus cicera*: outcomes from a linkage mapping approach. *Plant Dis.* 104 2875–2884. 10.1094/PDIS-03-20-0513-RE 32954987

[B51] Schaefer H. Hechenleitner P. Santos-Guerra A. Menezes de Sequeira M. Pennington R. T. Kenicer G. (2012). Systematics, biogeography, and character evolution of the legume tribe Fabeae with special focus on the middle Atlantic island lineages. *BMC Evol. Biol.* 12:250. 10.1186/1471-2148-12-250 23267563 PMC3547781

[B52] Sillero J. C. Fondevilla S. Davidson J. Vaz Patto M. C. Warkentin T. D. Thomas J. (2006). Screening techniques and sources of resistance to rusts and mildews in grain legumes. *Euphytica* 147 255–272. 10.1007/s10681-006-6544-1

[B53] Smýkal P. Kenicer G. Flavell A. Corander J. Kosterin O. Redden R. (2011). Phylogeny, phylogeography and genetic diversity of the *Pisum* genus. *Plant Genet. Res.* 9 4–18. 10.1017/S147926211000033X

[B54] Stakman E. C. Stewart D. M. Loegering W. Q. (1662). *Identification of Physiologic Races of Puccinia Graminis Var Tritici.* United States: CABI.

[B55] Stackman E. C. Stewart D. M. Loegering W. Q. (1962). Identification of physiologic races of *Puccinia graminis var. tritici*. Washington, DC: USDA, Agricultural Research Service, E617.

[B56] Swanson-Wagner R. A. Decook R. Jia Y. Bancroft T. Ji T. Zhao X. (2009). Paternal dominance of trans-eQTL influences gene expression patterns in maize hybrids. *Science* 326 1118–1120. 10.1126/science.1178294 19965432

[B57] Thomas B. R. Romero G. O. Nevins D. J. Rodriguez R. L. (2000). New perspectives on the endo-beta-glucanases of glycosyl hydrolase Family 17. *Int. J. Biol. Macromol.* 27 139–144. 10.1016/S0141-8130(00)00109-4 10771063

[B58] Toyoda K. Kawahara T. Ichinose Y. Yamada T. Shiraishi T. (2000). Potentiation of phytoalexin accumulation in elicitor-treated epicotyls of pea (*Pisum sativum*) by a diacylglycerol kinase inhibitor. *J. Phytopathol.* 148 633–636. 10.1111/J.1439-0434.2000.00568.X

[B59] Van Aken O. Zhang B. Carrie C. Uggalla V. Paynter E. Giraud E. (2009). Defining the mitochondrial stress response in *Arabidopsis thaliana*. *Mol. Plant* 2 1310–1324. 10.1093/MP/SSP053 19995732

[B60] Van Ooijen J. W. (2009). *MapQTL 6, Software for the Mapping of Quantitative Trait Loci in Experimental Populations of Diploid Species.* Wageningen: Kyazma B. V.

[B61] Vaz Patto M. C. Fernández-Aparicio M. Moral A. Rubiales D. (2009). Pre and posthaustorial resistance to rusts in *Lathyrus cicera* L. *Euphytica* 165 27–34. 10.1007/s10681-008-9737-y

[B62] Vaz Patto M. C. Rubiales D. (2009). Identification and characterization of partial resistance to rust in a germplasm collection of *Lathyrus sativus* L. *Plant Breed.* 128 495–500. 10.1111/j.1439-0523.2008.01601.x

[B63] Vaz Patto M. C. Rubiales D. (2014). *Lathyrus* diversity: available resources with relevance to crop improvement – *L. sativus* and *L. cicera* as case studies. *Ann. Bot.* 113 895–908. 10.1093/aob/mcu024 24623333 PMC3997641

[B64] Vaz Patto M. C. Skiba B. Pang E. C. K. Ochatt S. J. Lambein F. Rubiales D. (2006). *Lathyrus* improvement for resistance against biotic and abiotic stresses: From classical breeding to marker assisted selection. *Euphytica* 147 133–147. 10.1007/s10681-006-3607-2

[B65] Voorrips R. E. (2002). MapChart: software for the graphical presentation of linkage maps and QTLs. *J. Hered.* 93 77–78. 10.1093/JHERED/93.1.77 12011185

[B66] Võsa U. Claringbould A. Westra H. J. Bonder M. J. Deelen P. Zeng B. (2021). Large-scale cis- and trans-eQTL analyses identify thousands of genetic loci and polygenic scores that regulate blood gene expression. *Nat. Genet.* 53 1300–1310. 10.1038/s41588-021-00913-z 34475573 PMC8432599

[B67] Wallace J. G. Larsson S. J. Buckler E. S. (2013). Entering the second century of maize quantitative genetics. *Heredity* 112 30–38. 10.1038/hdy.2013.6 23462502 PMC3860165

[B68] Wang G. Kong H. Sun Y. Zhang X. Zhang W. Altman N. (2004). Genome-wide analysis of the cyclin family in *Arabidopsis* and comparative phylogenetic analysis of plant cyclin-like proteins. *Plant Physiol.* 135 1084–1099. 10.1104/PP.104.040436 15208425 PMC514142

[B69] Wang J. Yu H. Xie W. Xing Y. Yu S. Xu C. (2010). A global analysis of QTLs for expression variations in rice shoots at the early seedling stage. *Plant J.* 63 1063–1074. 10.1111/j.1365-313X.2010.04303.x 20626655

[B70] Wang X. Chen Q. Wu Y. Lemmon Z. H. Xu G. Huang C. (2018). Genome-wide analysis of transcriptional variability in a large maize-teosinte population. *Mol. Plant* 11 443–459. 10.1016/J.MOLP.2017.12.011 29275164

[B71] Wu L. Fredua-Agyeman R. Hwang S.-F. Chang K.-F. Conner R. L. McLaren D. L. (2021). Mapping QTL associated with partial resistance to *Aphanomyces* root rot in pea (*Pisum sativum* L.) using a 13.2 K SNP array and SSR markers. *Theor. Appl. Genet.* 134 2965–2990. 10.1007/S00122-021-03871-6 34129066

[B72] Xing H. Fu X. Yang C. Tang X. Guo L. Li C. (2018). Genome-wide investigation of pentatricopeptide repeat gene family in poplar and their expression analysis in response to biotic and abiotic stresses. *Sci. Rep.* 8:2817. 10.1038/s41598-018-21269-1 29434322 PMC5809412

[B73] Zegeye H. Rasheed A. Makdis F. Badebo A. Ogbonnaya F. C. (2014). Genome-wide association mapping for seedling and adult plant resistance to stripe rust in synthetic hexaploid wheat. *PLoS One* 9:e105593. 10.1371/JOURNAL.PONE.0105593 25153126 PMC4143293

