# Supplementary figures and images for: Integrating Phenotypic and Gene Expression Linkage Mapping to Dissect Rust Resistance in Chickling Pea
Source: Front Plant Sci. 2022 Apr 7;13:837613. doi: 10.3389/fpls.2022.837613 (PMC9021875; doi:10.3389/fpls.2022.837613)

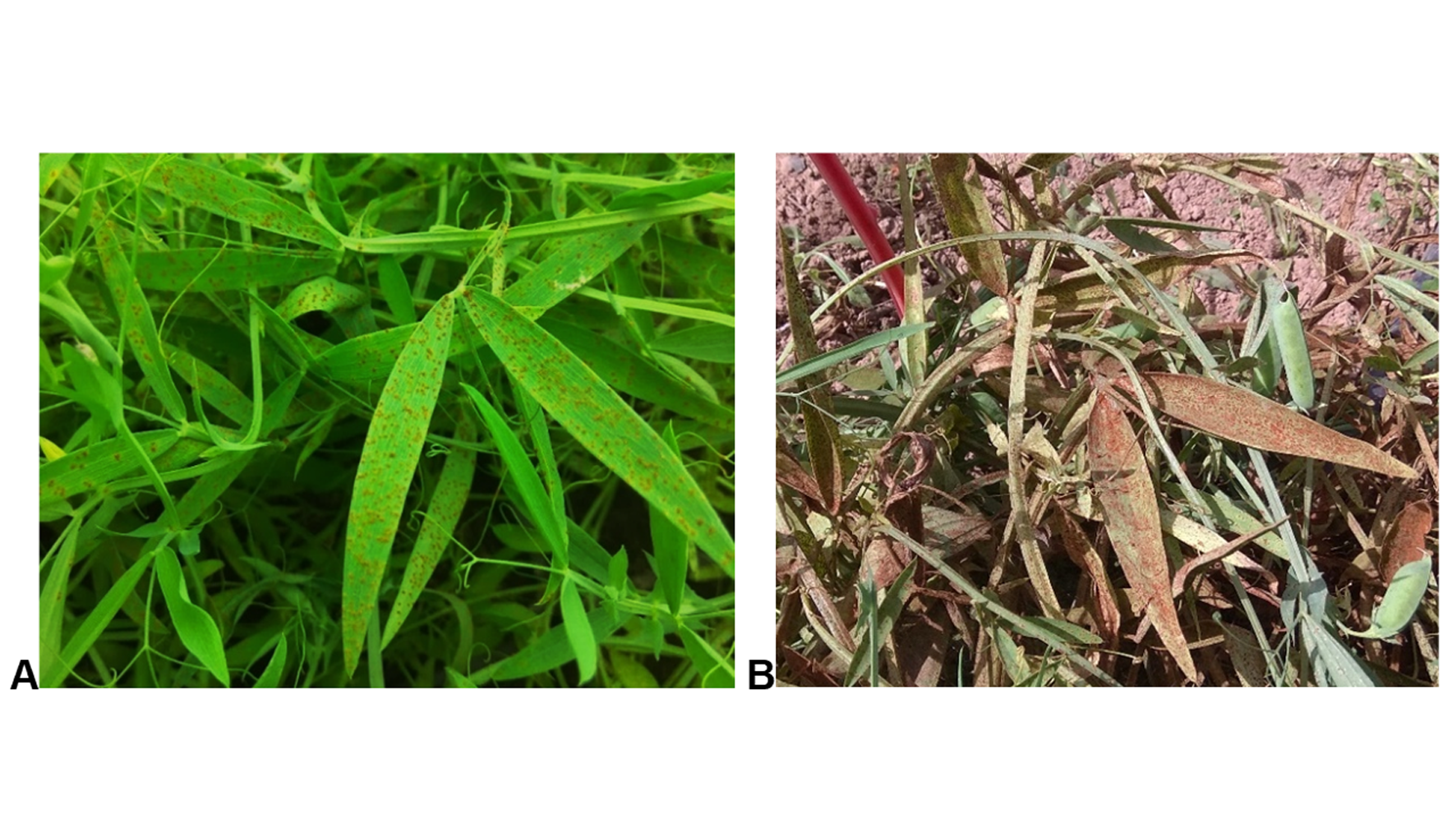

Supplement: Supplementary Figure 1 — Leaves of Lathyrus cicera inoculated with Uromyces pisi, showing a compatible reaction between plant and pathogen (IT = 4), (A) under controlled conditions (growth chamber) 11 days after inoculation with U. pisi and (B) under semi-controlled field conditions 3 months after inoculation with U. pisi. [file Image_1.TIF]

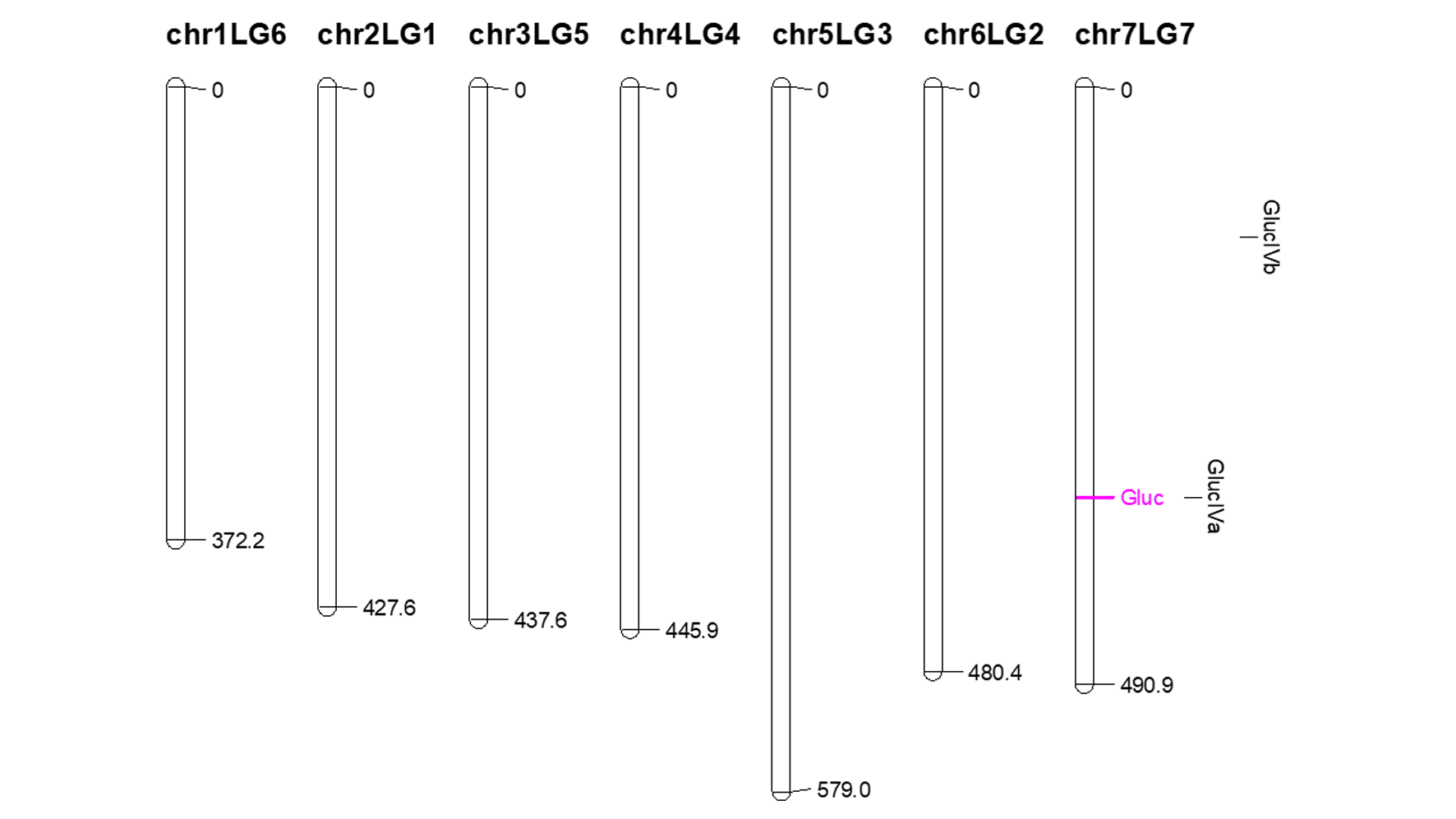

Supplement: Supplementary Figure 2 — Expression QTLs syntenic regions of Lathyrus cicera recombinant inbred lines population (BGE023542 × BGE008277) for rust (Uromyces pisi) response mapped on the physical map of Pisum sativum. Physical distances given in Mbp are indicated for each P. sativum chromosome. Horizontal pink line indicates the syntenic position of L. cicera differentially expressed gene in P. sativum chromosomes. Black horizontal lines represent the syntenic regions of L. cicera eQTL intervals mapped on P. sativum genome. [file Image_2.TIF]
